# Comparison of the MyoRing implantation depth by mechanical dissection using PocketMaker microkeratome versus Melles hook via AS-OCT

**DOI:** 10.1186/s12886-018-0806-2

**Published:** 2018-06-07

**Authors:** Shiva Pirhadi, Neda Mohammadi, Seyed Aliasghar Mosavi, Hashem Daryabari, Hossein Aghamollaei, Khosrow Jadidi

**Affiliations:** 1 Department of Biomedical Engineering, Tehran Science and Research Branch, Islamic Azad University, Tehran, Iran; 2 Department of Epidemiology and Biostatistics, School of Public Health, Tehran University of Medical Sciences, Tehran, Iran; 3 Vision Health Research Center, Semnan University of Medical Sciences, Semnan, Iran; 4 Department of Ophthalmology, Baqiyatallah University of Medical Sciences, Tehran, Iran

**Keywords:** Cornea, Keratoconus, Intracorneal rings, PocketMaker microkeratome, Melles hook

## Abstract

**Background:**

This paper seeks to evaluate the depth and outcomes of MyoRing implantation using two mechanical dissection procedures including: PocketMaker microkeratome in opposition to the Melles hook method.

**Methods:**

This retrospective study was carried out on 39 eyes of 38 keratoconus patients (28 male and 10 female) with the mean age of $28.97\frac{+}{.}10.37$ years and had undergone MyoRing implantation by the two mentioned methods. The MyoRing was inserted into the corneal pocket which was made manually in 18 eyes (Melles hook group) or with PocketMaker microkeratome in 21 eyes (PocketMaker group). The mean follow up time was $9.81\frac{+}{.}3.7$ months with pre-operative and post-operative ophthalmic examination including uncorrected visual acuity (UCVA), best-corrected visual acuity (BCVA), keratometry readings and central corneal thickness measurement. AS-OCT (Casia, SS-1000, Tomey, Nagoya, Japan) imaging was used to measure MyoRing insertion depth, exactly.

**Results:**

Pre-operative and post-operative UCVA (LogMAR) mean change for the PocketMaker and Melles hook groups were recorded at 0.75 ± 0.32 and 0.78 ± 0.33, respectively. Similarly, BCVA (LogMAR) mean change were 0.27 ± 0.22 and 0.23 ± 0.22. Mean keratometry (Kmean) change were 6.06 ± 4.18 and 6.56 ± 3.55 respectively. UCVA change (*P* = 0.767), BCVA change (*P* = 0.77) and Kmean change (*P* = 0.693) showed that there was no statistically significant difference between both groups for any parameter. Depth measurements achieved from AS-OCT images showed that there was no statistically significant difference in pocket depth between two methods of MyoRing implantation (*P* = 0.413).

**Conclusions:**

The results of Myoring implantation outcomes using mechanical dissection via PocketMaker microkeratome as against Melles hook are comparable.

## Background

Keratoconus is a non-inflammatory disease of the cornea and it manifests by progressive steepening, thinning and ectasia of the cornea [1]. This condition negatively affects patient’s visual function. There are several ways to manage different stages of this disease. These stages include the use of contact lens, corneal collagen cross-linking (CXL), intracorneal ring implantation, lamellar and penetrating keratoplasty [2–6].

The intracorneal ring is made of synthetic material that can be inserted into the corneal stroma to reshape the cornea. Cornea remodeling, using this device can result to modification of cornea curvature and improvement of visual acuity [7]. Intraocular corneal rings available in the market include incomplete and complete rings. The incomplete rings include: Intacs (Addition Technology Inc.), Ferrararings (Ferrara Ophthalmic Ltd.), and Keraring (Mediphacos Ltd.). MyoRing (Dioptex GmbH, Austria), as a complete ring, is a new method that can be safe and effective in the treatment of Keratoconus [7–12]. The ring is inserted into an intrastromal pocket, which is created by femtosecond laser [11, 13] or a microkeratome PocketMaker (Dioptex GmbH, Austria) [8]. It can also be inserted mechanically using the Melles hook approach. In previous studies, the depth of the corneal pocket was suggested to be 300 μm [11, 12].

Different depths of ring insertion may have different visual outcomes. The implantation depth of intracorneal ring has been measured by scheimpflug [14, 15] or anterior segment optical coherence tomography (AS-OCT) images in previous studies [16–19]. In some of these studies, actual versus intended insertion depth were assessed whereas others evaluated visual outcomes relative to intracorneal ring depth. In addition, pocket creation for intracorneal ring implantation using femtosecond laser and PocketMaker has been compared in several studies [20–23]. However, to the best of our knowledge, there are no reports on the MyoRing implantation depth measurement using AS-OCT images and comparison of its insertion by two methods including PocketMaker and Melles hook. This study fixed MyoRing at a depth of 300 μm by these two different methods. Thereafter, the exact inserted depth was determined using high-resolution AS-OCT postoperatively to determine if the MyoRing was implanted at the same depth by the two methods. Also, a comparison was made between the visual and refractive outcomes of MyoRing implantation by PocketMaker microkeratome against the Melles hook method.

## Methods

In this study, 39 eyes of 38 keratoconus patients were registered between July 2011 to April 2015 at Bina Eye Hospital, Tehran. The inclusion criteria involves factors such as patients with keratoconus, poor visual acuity with glasses, contact lens intolerance, a clear central cornea, a minimum corneal thickness of 400 μm, and a maximum keratometry reading of less than 60 diopters (D). Keratoconus grading was determined based on the Krumeich classification [24]. MyoRing is available in 5- or 6-mm both in diameter and in thickness ranging from 200 to 320 μm (in 20 μm increments). The appropriate MyoRing was selected based on an innovative nomogram, previously described in detail [25]. The Corneal pocket was made using PocketMaker microkeratome in 21 eyes and manually using Melles hook in 18 eyes. The eyes were assigned for pocket creation by PocketMaker microkeratome or Melles hook based on the corneal steep meridian. The PocketMaker method was employed if the steep meridian was in the temporal area in the range of − 30 to +30 degrees. If the steep meridian was out of this range or in the superior area, the mechanical method would be selected. All patients were required to sign an informed consent form before treatment upon explanation of the purpose and procedures of the surgery. Afterwards, written informed consent forms were obtained from all participants.

All procedures performed in this study are in accordance with the ethical standards of the institutional research committee and with the 1964 Helsinki declaration and its later amendments.

### Surgical procedure

All surgical procedures were performed by the same experienced surgeon (Kh.J) in a general operating room using a surgical microscope under topical anesthesia with 0.5% proparacaine hydrochloride solution. MyoRing implantation in all surgeries was performed by the steep meridian. In order to mark the steep point of the cornea, keratometry and keratoscopy were used in a sterilized situation.

### Mechanical dissection using PocketMaker microkeratome

Mechanical dissection using PocketMaker microkeratome procedure for MyoRing implantation included the creation of pocket within corneal stroma at 8 or 9 mm diameter (based on MyoRing diameter) and 300 μm in depth using a PocketMaker microkeratome (Dioptex GmbH). After determining the correct position of the blade, the micro-vibrating diamond blade was set at 300 μm of the measured corneal thickness and a single 0.5 mm radial incision was made at the steepest meridian. Thereafter, the ring was inserted into the created pocket and its position was adjusted intraoperatively using a keratoscope.

### Mechanical dissection using Melles hook

Mechanical dissection using the Melles hook procedure of Myoring implantation involved making an incision in the steep axis of the cornea with 0.5 mm length (smaller than MyoRing diameter) using a diamond blade. Afterward, a dissection in 300 μm depth with 3 mm (bigger than MyoRing diameter) was created using a Melles hook. Subsequently, the MyoRing was inserted into the pocket, which was mechanically created in the cornea, and its proper centration was determined using a keratoscope.

In both surgical methods, it was observed that the created pocket was capable of self-sealing and did not require any suture. Eventually, a bandage contact lens (Bausch & Lomb) was placed on the cornea and MyoRing implantation surgery was completed using chloramphenicol (Sinadaru Laboratories, Iran) eye drops. The bandage contact lens was removed 1 day after surgery. Four drops per day of chloramphenicol and betamethasone (Sinadaru Laboratories, Iran) and six drops per day of preservative-free artificial tears (Artelac Rebalance, Bausch & Lomb, Inc., USA) were prescribed postoperatively. The chloramphenicol drop was interrupted 1 week after surgery while the betamethasone dosage was tapered off during 4–6 weeks. Also, Artelac was continued 6 times a day for 1 month.

### Patient evaluation

Preoperative and postoperative ophthalmic examinations in PocketMaker and manual groups included uncorrected visual acuity (UCVA), best-corrected visual acuity (BCVA) with a standard Snellen chart, corneal thickness, thinnest corneal point, anterior chamber depth (ACD) and keratometry readings by scanning-slit topography (Orbscan II), manifest spherical and cylindrical refraction and spherical equivalent (SE). For statistical analysis, decimal Snellen UCVA and BCVA were converted to LogMAR values.

**Fig. 1:**
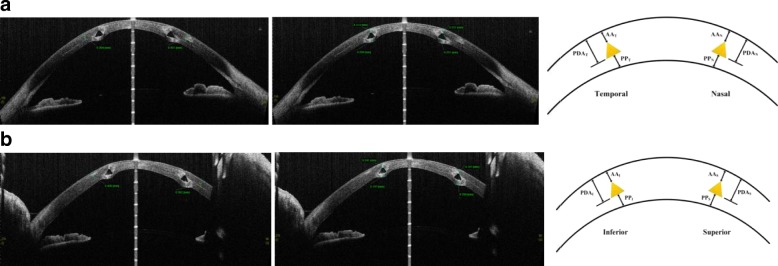
The distances between the pocket depth to the anterior corneal surface (Left), the anterior ring surface and the anterior corneal surface, the posterior ring surface and the posterior corneal surface (middle) and the schematics of AS-OCT images (Right) in horizontal (**a**) and vertical axes (**b**) after MyoRing implantation. In each axis 3 parameters defined: distances between the anterior ring surface and the anterior corneal surface (AA), the posterior ring surface and the posterior corneal surface (PP) and distances between the pocket depth and the anterior corneal surface (PDA). These parameters measured for temporal and nasal, inferior and superior sites for 0–180 and 90–270 axes, respectively

After surgery, AS-OCT (Casia, SS-1000, Tomey, Nagoya, Japan) images were used to measure MyoRing insertion depth, exactly. AS-OCT imaging was performed by one technician. The MyoRing depth and the depth of the intracorneal pocket were measured in horizontal and vertical axes at temporal, nasal, inferior and superior positions. Figure 1 shows 12 measured distances in two axes at 4 different sites in AS-OCT images. In each axis, distances between the anterior ring surface and the anterior corneal surface (AA), the posterior ring surface and the posterior corneal surface at ring sites (PP) and distances between the pocket depth and the anterior corneal surface (PDA) were measured at pocket sites (Fig. 1). Since an intracorneal ring was inserted into the cornea, it usually compressed the corneal lamella. Therefore, a decision was made to measure the PDA distance as the depth of the created pocket. For evaluating the MyroRing depth, nine parameters were defined as follows:

1. AA_0–180_ = (AA_T_ + AA_N_)/2

Mean of AA distance at temporal and nasal sites in the 0–180 axis.

2) AA_90–270_ = (AA_S_ + AA_I_) /2

Mean of AA distance at superior and inferior sites in the 90–270 axis.

3) AA_total_ = (AA_0–180_ + AA_90–270_) / 2

Mean of AA distance in the horizontal and vertical axes.

4) PP_0–180_ = (PP_T_ + PP_N_) /2

Mean of PP distance at temporal and nasal sites in the 0–180 axis.

5) PP_90–270_ = (PP_S_ + PP_I_) /2

Mean of PP distance at superior and inferior sites in the 90–270 axis.

6) PP_total_ = (PP_0–180_ + PP_90–270_) / 2

Mean of PP distance in horizontal and vertical axes.

7) PDA _0–180_ = (PDA_T_ + PDA_N_) /2

Mean of PDA distance at temporal and nasal sites in the 0–180 axis.

8) PDA_90–270_ = (PDA_S_ + PDA_I_) /2

Mean of PDA distance at superior and inferior sites in the 90–27 axis.

9) PDA_total_ = (PDA_0–180_ + PDA_90–270_) /2

Mean of PDA distance in the horizontal and vertical axes.

All distances with 0–180 index compared with the same ones in the 90–270 axis intragroup for PocketMaker and manual groups. The total indices of other parameters were compared between groups.

### Statistical analysis

Statistical analysis was performed using SPSS software for Windows (version 24; SPSS Inc., Chicago, IL, USA). To compare the same distances at different axes in each group, the t-paired test was used. The t-test or if necessary, its replace test, nonparametric Mann-Whitney, was used to compare the depth parameters with total indices between two groups. The paired t-test was used to compare visual, refractive, keratometric and corneal thickness variables before and after ring insertion by the method of insertion used; and the non-parametric Wilcoxon test was used in its place wherever required. The two groups were compared in terms of these variables before ring implantation using the independent t-test, and the non-parametric Mann-Whitney test (where necessary). After ring implantation, these variables were also compared between groups using the independent t-test and the non-parametric Mann-Whitney test (where necessary). The difference between these variables before and after ring insertion was measured as the mean changes, and the independent t-test was used to compare the mean changes obtained by the Melles hook method and the PocketMaker mikrokeratome.

## Results

This study evaluated 39 eyes of 38 keratoconus patients with a mean age of $28.97\frac{+}{.}10.37$years (Range 17–55 years). The Corneal pocket was made using PocketMaker in 21 eyes and manually using Melles hook in 18 eyes. The mean follow-up period was$\kern0.5em 9.81\frac{+}{.}3.7$months in the PocketMaker group and$9.89\frac{+}{.}3.3$ months in the Melles hook group. MyroRing with 5- and 6-mm diameter were fixed at 12 and 9, 10 and 8 eyes in the PocketMaker and Melles hook groups, respectively. The mean MyoRing thickness was $298.1\frac{+}{.}36.27$ and $302.22\frac{+}{.}36.87$ in the PocketMaker and Melles hook groups, respectively. There were no statistically significant differences between 2 groups in the mean follow-up (*P* > 0.999), patients’ ages (*P* = 0.6) and ring thickness (*P* = 0.41). Moreover, preoperative keratoconus grades were determined in each group (Table 1). No significant difference was found in the distribution of grades between both groups (*P* = 0.715, chi-square test).

**Table 1:** Keratoconus grading based on Krumeich classification

Grade of KCN	Eyes, n (%)	
	Mechanical	PocketMaker
*1*	3 (16)	6 (28)
*2*	7 (38)	9 (42)
*3*	1 (5)	1 (4)
*4*	7 (38)	5 (23)

All AA, PP and PDA depth parameters were compared between the 0–180 and 90–270 axes in each group (Table 2). There were no statistically significant differences in depth parameters between the two axes in the PocketMaker group. In the Melles hook group, only PP parameter had a significant difference between the two axes (*P* = 0.001) which means that the corneal thickness below the ring in the 0–180 axis is lower compared to the other axis. The parameters with total indices between groups were also compared (Table 3). There were no significant differences in these variables. Moreover, a correlation analysis was performed to evaluate the effect of MyoRing thickness on depth parameters. No correlation was found between MyoRing thickness and *AA*_*total*_ (*r* = − 0.11, *P* = 0.636), *PP*_*total*_ (*r* = 0.011, *P* = 0.961), and *PDA*_*total*_(*r* = − 0.031, *P* = 0.892) parameters in the PocketMaker group. Also, there were no correlations between Myoring thickness and *AA*_*total*_ (*r* = − 0.156, *P* = 0.536), *PP*_*total*_ (*r* = 0.027, *P* = 0.914), and *PDA*_*total*_ (*r* = − 0.026, *P* = 0.918) parameters in the mechanical group.

**Table 2:** Comparison of depth parameters at horizontal and vertical axes between Manual group and PocketMaker group

Variables	Type of surgery					
	Mechanical			PocketMaker		
	N	Mean ± SD	*P*-value	N	Mean ± SD	*P*-value
*AA*_0 − 180_	18	206.33 ± 48.68	0.07	21	199.14 ± 36.77	0.227
*AA*_90 − 270_	18	196.22 ± 49.83		21	216.95 ± 66.59	
*PP*_0 − 180_	18	208.86 ± 33.76	0.001	21	230.64 ± 54.87	0.364
*PP*_90 − 270_	18	226.64 ± 38.4		21	234.81 ± 51.02	
*PDA*_0 − 180_	18	341.14 ± 42.58	0.447	21	347.6 ± 36.19	0.548
*PDA*_90 − 270_	18	334.58 ± 53.15		21	351.38 ± 52.45	

AA_0–180_: Mean of AA distance at temporal and nasal sites in 0–180 axis, AA_90–270_: Mean of AA distance at superior and inferior sites in 90–270 axis, PP_0–180_: Mean of PP distance at temporal and nasal sites in 0–180 axis, PP_90–270_: Mean of PP distance at superior and inferior sites in 90–270 axis, PDA _0–180_: Mean of PDA distance at temporal and nasal sites in 0–180 axis, PDA_90–270_: Mean of PDA distance at superior and inferior sites in 90–27 axis

**Table 3:** Comparison of depth total parameters between manual group and PocketMaker group

variables	Type of surgery	N	Mean ± SD	*P*-value
*AA*_*total*_	Manual	18	201.2778 ± 47.99	0.644
	PocketMaker	21	208.0476 ± 42.68	
*PP*_*total*_	Manual	18	217.75 ± 34.96	0.307
	PocketMaker	21	232.7262 ± 51.97	
*PDA*_*total*_	Manual	18	337.8611 ± 44.72	0.413
	PocketMaker	21	349.4881 ± 42.76	

The visual, refractive, keratometric, corneal thickness and ACD variables were assessed before and after the operation in each group and compared between the two groups (Table 4). The results showed a significant improvement in UCVA, BCVA, sphere, cylinder, SE, Sim-K, astigmatism as well as the maximum, minimum and mean keratometry in each group. Corneal thickness and the thinnest corneal point increased in both groups after the operation, and this increase was significant in the Melles hook group. ACD reduced after the operation in both groups, but not significantly. There were no significant differences between the two groups in these variables before the operation. However, after the operation, the two groups differed significantly only in terms of the cylinder variable.

**Table 4:** Comparison of refractive, keratometric, thickness and visual outcomes between manual group and PocketMaker group

Variables	Mean ± SD		
	Manual	PocketMaker	P-value
UCVA (*LogMAR*)			
*Pre*	1.18 ± 0.25	1.03 ± 0.28	0.083
*Post*	0.39 ± 0.25	0.27 ± 0.17	0.106
*Mean change*	0.78 ± 0.33	0.75 ± 0.32	0.767
*P-value*	< 0.001	< 0.001	
BCVA (*LogMAR*)			
*Pre*	0.56 ± 0.34	0.48 ± 0.2	0.791
*Post*	0.26 ± 0.19	0.21 ± 0.13	0.512
*Mean change*	0.23 ± 0.22	0.27 ± 0.22	0.77
*P-value*	< 0.001	< 0.001	
Sphere (*D*)			
*Pre*	−7.89 ± 4.42	−7.5 ± 2.97	0.754
*Post*	−1.49 ± 3.46	−0.37 ± 1.6	0.394
*Mean change*	−6.4 ± 4.79	−7.13 ± 3.5	0.584
*P-value*	< 0.001	< 0.001	
Cylinder (*D*)			
*Pre*	−5.58 ± 2.1	−4.36 ± 1.33	0.053
*Post*	−2.65 ± 1.44	−1.46 ± 0.77	0.006
*Mean change*	−2.93 ± 2.55	−2.89 ± 1.46	0.954
*P-value*	< 0.001	< 0.001	
SE (*D*)			
*Pre*	−10.68 ± 4.26	−9.68 ± 2.93	0.407
*Post*	−2.82 ± 3.42	−1.1 ± 1.51	0.11
*Mean change*	−7.86 ± 4.7	−8.58 ± 3.51	0.59
*P-value*	< 0.001	< 0.001	
Sim k astigmatism(*D*)			
*Pre*	−5.81 ± 1.858	− 5.1 ± 2.56	0.336
*Post*	−2.54 ± 1.4642	−2.2 ± 1.46	0.364
*Mean change*	−3.26 ± 2.45	−2.89 ± 2.29	0.633
*P-value*	< 0.001	< 0.001	
Kmax (*D*)			
*Pre*	55.73 ± 5.43	53.14 ± 5.96	0.213
*Post*	47.84 ± 3.31	45.85 ± 3.14	0.192
*Mean change*	7.89 ± 4.14	7.29 ± 5.06	0.692
*P-value*	< 0.001	< 0.001	
Kmin (*D*)			
*Pre*	51.12 ± 5.54	48.48 ± 4.11	0.097
*Post*	45.34 ± 3.54	43.64 ± 2.85	0.106
*Mean change*	5.77 ± 4.81	4.83 ± 3.75	0.495
*P-value*	< 0.001	< 0.001	
Kmean (*D*)			
*Pre*	53.26 ± 5.05	50.81 ± 4.86	0.192
*Post*	46.56 ± 3.46	44.75 ± 2.91	0.174
*Mean change*	6.56 ± 3.55	6.06 ± 4.18	0.693
*P-value*	< 0.001	< 0.001	
Corneal thickness (*μm*)			
*Pre*	421.06 ± 45.58	452.52 ± 60.42	0.078
*Post*	447.78 ± 27.85	467.67 ± 44.61	0.099
*Mean change*	−26.72 ± 41.99	−15.14 ± 56.6	0.479
*P-value*	0.015	0.234	
Thinnest corneal point (*μm*)			
*Pre*	394.44 ± 57.76	431.67 ± 68.43	0.077
*Post*	425.33 ± 31.05	441.05 ± 45.8	0.226
*Mean change*	−30.89 ± 52.66	−9.38 ± 62.49	0.257
*P-value*	0.023	0.499	
ACD (mm)			
*Pre*	3.7106 ± 0.59	3.59 ± 0.48	0.508
*Post*	3.3975 ± 0.42	3.39 ± 0.39	0.773
*Mean change*	0.31 ± 0.49	0.21 ± 0.25	0.397
*P-value*	0.023	0.001	

*UCVA* Uncorrected visual acuity, *BCVA* Best-corrected visual acuity, *Sphere* manifest spherical refraction, *Cylinder* manifest cylindrical refraction, *SE* Spherical equivalent, *K* keratometric power, *ACD* Anterior chamber depth

## Discussion

The MyoRingintracorneal implantation has been presented in order to treat keratoconus as well as improve visual and refractive outcomes. Three techniques were suggested to make a pocket for MyoRing insertion. ThePocket can be created using a femtosecond laser [11, 13], using a PocketMaker microkeratome (Dioptex GmbH, Austria) [8] and manually, using the Melles hook approach. The present study is the first to compare the Melles hook method and the PocketMaker in terms of both the pocket depth created, the refraction as well as the visual outcomes.

Daxer et al. compared two methods of MyoRing implantation, including the Femtosecond laser-assisted method and the PocketMaker. They did not assess the pocket depth created by the two methods and thus was insufficient to compare their fraction and visual outcomes between the two groups [23].

The PocketMaker is an expensive device and its delicate blades may be damaged in the autoclaving process or during the operation, and repairing damaged blades requires a high expenditure of time and money. In manual ring insertion, only Melles hooks are required, which are inexpensive and easy to use. The present study was conducted to compare the creation of pockets using the PocketMaker and Melles hooks in terms of the depth of implant, refraction and visual outcomes in order to determine if the Melles hook method can be used in places where the PocketMaker is unavailable, provided the outcomes are similar. The MyoRing was therefore inserted to the same depth (300 μm) using these two different methods. Thereafter, Casia AS-OCT was used to measure the precise post-operative ring implantation depth created in the two methods. The post-operative ring implantation depth was measured in horizontal and vertical axes in temporal, nasal, superior and inferior positions.

In the present study, in the Melles hook group, the mean distance from the posterior part of the ring to the posterior part of the cornea was 208.86 ± 33.76 μm in the nasal and temporal regions and 226.64 ± 38.4 μm in the superior and inferior regions, which is statistically significant (*P* = 0.001). Nonetheless, no significant differences were observed in the PocketMaker group (*P* = 0.364). In the Melles hook group, the ring was implanted 18 μm deeper in the horizontal axis, and the uneven movement of the hook in the Melles hook method may have caused this difference. However, in the PocketMaker group, the eye pressure increased to 70 mmHg, and the incision was created on a smooth surface. Despite this difference, measuring the mean distance in all the four regions in the two groups and comparing it with each group showed no significant differences. In addition, the mean distance between the anterior part of the ring and the anterior part of the cornea was also measured in the four regions and no significant differences were observed between the two groups (*P* = 0.664). It can thus be concluded that the mean depth of ring implantation at the distance from the anterior part of the ring to the anterior part of the cornea and from the posterior part of the ring to the posterior part of the cornea is the same in both methods.

In one study, Koussai et al. compared Intacs implant depth in mechanical and Femtosecond laser-assisted methods using AS-OCT. The mean difference between the depth expected before the operation and the final implant depth was 76.64 μm in the mechanical group and 85.85 μm in the Femtosecond-assisted group, with no significant differences between the two groups. Their study showed that shallower Intacs implant depths had been created in both methods compared to the expected depth [20]. Gorgun et al. measured anterior stromal thickness from the Ferrara segment apex after ring implantation using a Femtosecond laser along with an AS-OCT and found that the Ferrara segments were implanted 97 μm shallower on average [16]. In another study, Barbara et al. measured the final implant depth and the expected depth after Intacs insertion in the mechanical method using an AS-OCT. It was discovered that the Intacsimplant depth created was 153 μm shallower than the expected depth [19]. In the present study, the pocket depths created in the PocketMaker and Melles hook groups were compared to the target depth of 300 μm. The measurements showed that the mean pocket depth created in the horizontal and vertical axes was 349.48 μm in the PocketMaker group and 337.86 μm in the Melles hook group. Although the depth created was 50 μm deeper in the PocketMaker group and 38 μm in the Melles hook group compared to the expected depth, no significant differences were observed between both groups (*P* = 0.413). Thus, both the PocketMaker and the Melles hook method can be said to have created similar pocket depths for MyoRing implantation.

The surgeon’s skill, however, is a noteworthy point. Although surgical skills are required to achieve the correct depth in ring implantation in both methods, these skills are twice as important as in the Melles hook method, because the surgeon’s lack of skills in the Melles hook method can lead to complications such as anterior or posterior corneal perforation, superficial ring placement and ring extrusion. In the present study, no intraoperative or postoperative complications were observed in either group.

As already discussed, an 18-μm difference was observed in the Melles hook group between the horizontal and vertical axes at the distance from the posterior part of the ring to the posterior part of the cornea, indicating uneven hook movement. Although this 18-μm difference can be overlooked, an uneven pocket can affect visual outcomes. The hypothesis in the present study was that the sharper and more regular pocket made by the PocketMaker compared to the Melles hook method, the better the visual outcomes of the PocketMaker. The two groups were therefore compared in terms of SE, UCVA, BCVA and keratometric parameters.

Daxer et al. compared refraction and visual outcomes after MyoRing implantation using a PocketMaker and the Femtosecond method and obtained similar outcomes in the two methods [23]. The present findings showed improvements in both groups in UCVA, BCVA, SE and keratometric parameters, with no significant differences between the two groups. It can thus be concluded that the created pocket did not affect the final outcomes in either method.

The limitations of this study include the small sample of treated eyes and the short follow up period. However, due to the difference in the indications of selected patients for MyoRing, and since only one study has assessed the two methods of MyoRing implantation (Daxer et al.), in which only 14 eyes were examined, it appears that the comparison of 39 eyes in the present study is reasonable.

## Conclusions

In conclusion, the MyoRing implant depths created by the PocketMaker and the Melles hook may be considered similar. On the other hand, if the surgeon is adequately skilled, the mechanical method using Melles hook can produce similar outcomes with fewer costs. However, large comparative multicenter studies are recommended to verify and further clarify these results.

## Abbreviations

AA: Distances between the anterior ring surface and the anterior corneal surface
ACD: Anterior chamber depth
AS-OCT: Anterior segment optical coherence tomography
BCVA: Best-corrected visual acuity
Kmax: Maximum keratometry reading
Kmean: Mean keratometry reading
Kmin: Minimum keratometry reading
LogMAR: Logarithm of the minimum angle of resolution
PDA: Distances between the pocket depth and the anterior corneal surface
PP: Distance between the posterior ring surface and the posterior corneal surface
*SD*: Standard deviation
SE: Spherical equivalent
UCVA: Uncorrected visual acuity
